# Effects of levodopa/carbidopa intestinal gel infusion on autonomic symptoms in advanced Parkinson’s disease: a systematic review

**DOI:** 10.1007/s10286-024-01090-9

**Published:** 2024-12-04

**Authors:** Silvia Galli, Lanfranco De Carolis, Edoardo Bianchini, Marika Alborghetti, Bianca Caliò, Pierre Pacilio, Alessandra Fanciulli, Francesco E. Pontieri, Domiziana Rinaldi

**Affiliations:** 1 Department of Neuroscience, Mental Health and Sensory Organs (NESMOS), Sapienza University of Rome, Via di Grottarossa, 1035, 00189 Rome, Italy; 2 Autonomy, Gerontology, E-Health, Imaging & Society (AGEIS), Université Grenoble Alpes, 38000 Grenoble, France; 3 Department of Neurology, Medical University of Innsbruck, Anichstrasse 35, 6020 Innsbruck, Austria

**Keywords:** Autonomic failure, Parkinson’s disease, Levodopa-carbidopa intestinal gel, Non-motor symptoms

## Abstract

**Purpose:**

Autonomic failure has a major impact on the quality of life of individuals with Parkinson’s disease (PD), especially in advanced stages of the disease. Levodopa/carbidopa intestinal gel (LCIG) infusion is a well-established treatment for advanced PD with severe motor fluctuations and provides substantial benefit in managing some non-motor symptoms (NMS), such as sleep, fatigue, and neuropsychiatric issues. The effect of LCIG on autonomic symptoms is by contrast not well known. Here we performed a systematic review on the influence of LCIG therapy on autonomic dysfunction in PD individuals.

**Methods:**

Following the PRISMA guidelines, we systematically searched for studies that included autonomic outcome measures in LCIG-treated PD individuals, limiting the search to articles written in English and published between January 2005 and June 2023. We evaluated improvement, stability, or worsening of gastrointestinal, urinary, and cardiovascular symptoms at six different timepoints according to clinimetric scale changes compared to baseline. Data on autonomic adverse events (AEs) possibly related to LCIG treatment were also collected.

**Results:**

Of the 1476 studies identified in the initial search, 16 ultimately met the inclusion criteria and underwent quality assessment and data extraction, with data from 1361 PD patients (18.3 months mean follow-up). Thirteen studies reported improvement or stability of gastrointestinal, urinary, and cardiovascular symptoms over the interventional period. One study found a worsening of cardiovascular symptoms and two of urological symptoms. Regarding safety, seven studies reported gastrointestinal (8.4%), urinary (0.5%), and cardiovascular (1.1%) autonomic LCIG-related AEs.

**Conclusions:**

LCIG infusion may help to reduce the burden of autonomic symptoms in advanced PD. Prospective studies specifically addressing the effect of LCIG on autonomic function in advanced PD are warranted.

**Supplementary Information:**

The online version contains supplementary material available at 10.1007/s10286-024-01090-9.

## Introduction

Parkinson’s disease (PD) is a chronic progressive neurodegenerative disorder of unknown etiology, clinically characterized by bradykinesia, rigidity, postural instability, and resting tremor [1]. Beyond these cardinal motor symptoms, individuals with PD frequently experience non-motor symptoms (NMS), including sensory, neuropsychiatric, cognitive, sleep, and autonomic disturbances. Most frequently, PD individuals suffer from gastrointestinal, urinary, and cardiovascular autonomic symptoms [2].

Gastrointestinal features in PD include excessive drooling, difficulty in swallowing, delayed gastric emptying, small intestine bacterial overgrowth, constipation, and defecatory dysfunction [2]. Gastrointestinal autonomic dysfunction may develop during the early, even premotor disease stage and generally worsens with disease progression. In addition to subjective discomfort, altered motility of the gastrointestinal tract may hamper the therapeutic efficacy of oral antiparkinsonian drugs, in particular levodopa [3]. Indeed, delayed or unpredictable gastric emptying and levodopa competition with dietary amino acids represent key risk factors for PD individuals to develop levodopa fluctuations [4].

Urinary symptoms are identifiable in up to 90% of individuals with PD [5] and can be attributed to either non-neurogenic (perineal laxity in women, prostatic hypertrophy or surgery in men) or neurogenic causes, which often coexist in older individuals. Urinary symptoms can be categorized into storage abnormalities (frequency, urgency, incontinence, and nocturia) that are mainly due to bladder detrusor overactivity, and voiding abnormalities (hesitancy, poor stream, urinary retention), which are primarily caused by detrusor-sphincter dyssynergia and develop, overall, less frequently in PD than in atypical parkinsonian disorders [5].

The most common manifestation of cardiovascular autonomic failure is orthostatic hypotension (OH), defined as a systolic blood pressure (BP) fall ≥ 20 mmHg or diastolic BP ≥ 10 mmHg within 3 min upon standing or head-up tilt [2]. OH is often accompanied by postprandial hypotension as well as supine hypertension (SH) [6]. Cardiovascular autonomic failure may sometimes precede the development of PD motor symptoms [7], but its frequency and severity increase over time, ultimately affecting every third PD individual [8]. Beyond symptomatic distress, cardiovascular autonomic failure plays a negative prognostic role in PD and represents a risk factor for cognitive impairment and increased chronic cerebrovascular damage [9].

Altogether, symptoms of autonomic failure severely affect the quality of life (QoL) and autonomy of individuals with PD, increase the caregiver burden, and tend to become more severe at advanced disease stages [10, 11] and in the case of excessive peripheral dopaminergic stimulation produced by plasmatic peaks of antiparkinsonian drugs [12]. In fact, oral levodopa administration has been shown to induce a hypotensive effect in patients with neurodegenerative parkinsonism, with a higher risk of overt levodopa-induced OH in those with underlying autonomic failure [13]. The pathophysiological mechanisms underlying the levodopa hypotensive effect are not fully understood, but likely include vasodilatation of the kidney and of the peripheral vascular bed due to decreased vascular sympathetic outflow and increased diuresis [13].

Almost 50 years after its introduction, levodopa remains the gold standard for treating PD motor symptoms. Continuous infusion of levodopa/carbidopa intestinal gel (LCIG) into the proximal jejunum via percutaneous endoscopic gastrojejunostomy (PEG-J) is a well-established therapy that leads to a more stable plasmatic levodopa concentration and significantly reduces the “OFF” time and “ON” time with troublesome dyskinesia in advanced PD cases [14], ultimately improving motor and non-motor fluctuations, the QoL and independence of affected individuals, as well as the caregiver burden [14–17].

Several interventional and observational studies have investigated the effect of LCIG on NMS, including autonomic ones. The the impact of LCIG on autonomic symptoms however remains poorly understood to date. For this reason, we performed a systematic review of the literature to appraise the influence of LCIG on symptoms of  gastrointestinal, urinary and cardiovascular autonomic failure in individuals with advanced PD.

## Materials and methods

### Search method

We conducted a systematic review following the Preferred Reporting Items for Systematic Reviews and Meta-analyses (PRISMA) guidelines on the available evidence of efficacy and safety outcomes of LCIG therapy on symptoms of autonomic failure in PD patients.

 We first searched the PubMed, Embase, Cochrane, and MEDLINE databases for relevant articles using the following keywords: [“Parkinson disease” OR “Parkinson’s disease” OR “Parkinson”] AND [“Intrajejunal levodopa infusion” OR “Levodopa carbidopa intestinal gel” OR “Levodopa carbidopa intrajejunal infusion” OR “LCIG” OR “Percutaneous endoscopic gastrostomy” OR “Levodopa intestinal infusion” OR “Levodopa infusion”] AND [“Dysautonomia” OR “Autonomic Dysfunction” OR “Autonomic failure” OR “Cardiovascular disease” OR “Non motor symptoms” OR “Bladder” OR “Urinary” OR “Urinary symptoms” OR “Nocturia” OR “Incontinence” OR “Urgency” OR “Urinary retention” OR “Gastrointestinal disease” OR “Dysphagia” OR “Gastroparesis” OR “Constipation” OR “Defecatory dysfunction” OR “Orthostatic hypotension” OR “Postprandial hypotension” OR “Supine hypertension” OR “Circadian blood pressure rhythm” OR “Nocturnal hypertension”].

Original articles published in peer-reviewed journals between January 2005 and June 2023 were considered. Only articles published in the English language on human subjects were included. Studies eligible for inclusion were randomized controlled trials (RCTs) and observational cohort studies, with or without a control group. Case reports, letters to the editor, reviews, meta-analyses, abstracts, editorials, case studies, and book chapters were excluded.

Studies had to meet the following criteria to be included: (1) participants were diagnosed with PD and treated with LCIG; (2) efficacy and safety outcomes regarding autonomic failure (gastrointestinal and/or urinary and/or cardiovascular symptoms) were assessed using validated clinical scales or in a descriptive manner.

Studies were excluded if they: (1) included individuals with diagnoses other than PD; (2) did not provide information on the statistical significance of the improvement, stability, or worsening of gastrointestinal, urinary, or cardiovascular autonomic symptoms at the different timepoints in comparison to baseline; (3) did not clearly report the considered timepoints of symptoms evaluation. No restrictions regarding the patient’s clinical/demographic features were applied.

Abstracts were screened for thematic relevance. After the exclusion of duplicated and non-relevant articles, full-text articles were independently reviewed for eligibility criteria by two authors (SG, LDC). In case of disagreement, at least two senior authors (AF, FEP, DR) were consulted to achieve consensus. The reference list of each included article was searched to screen for additional studies not captured by the original search strategy. Two further studies [16, 18], published after the end of the search period, were identified by senior authors (DR and FEP) and included because they were considered highly relevant for the purpose of the present work.

### Methodological quality

Two authors (SG, LDC) independently performed the quality appraisal of the selected studies. The risk of bias in individual studies was evaluated using the National Heart, Lung, and Blood Institute tools (NHLBI Quality Appraisal Tools) following the Cochrane Handbook recommendations [19]. These two authors rated the studies for their risk of bias (i.e., poor, fair, or good) using the recommended questionnaire-based analysis regarding study design, research question, sample size and selection, eligibility criteria, literature search, clarity of exposition, clarity of methods, description of results, potential confounding variables, and limitations [19].

### Data extraction

Baseline evaluation (T0) was defined as the last visit prior to the initiation of LCIG treatment. At T0, we collected data on the patient’s clinical and demographic features, Unified Parkinson’s Disease Rating Scale part III (UPDRS-III), and clinical rating scale scores for the assessment of gastrointestinal, urinary, and cardiovascular symptoms.

Clinical features and scores of rating scales, safety outcomes in terms of reported occurrence of adverse events (AEs) within the autonomic domains, and number of hours of LCIG infusion per day were also collected at six different timepoints after beginning of the LCIG therapy: < 6 months (T1), 6 months (T2), 12 months (T3), 12–24 months (T4), 24–36 months (T5), and > 36 months (T6). Improvement, stability, or worsening of gastrointestinal, urinary, and cardiovascular symptoms were evaluated at each timepoint, according to change in prevalence or, when available, the clinical rating scale score compared to T0.

The effect of LCIG on gastrointestinal symptoms was assessed through the Non-Motor Symptoms Scale (NMSS) domain 6 (sialorrhea, dysphagia, and constipation), Movement Disorders Society Unified Parkinson's Disease Rating Scale (MDS-UPDRS)-I item 11 (constipation), and constipation prevalence. For urinary symptoms, we used the NMSS domain 7 (urinary urgency, increased urinary frequency, and nocturia), UPDRS-I item 10 (general urinary problems), and urinary symptoms prevalence, severity, and frequency. For assessing the effect of LCIG on cardiovascular symptoms, we used the NMSS domain 1 (assessing the presence of light-headedness, dizziness, weakness on standing from sitting or lying position, falls or fainting), UPDRS-I item 12 (lightheadedness on standing), Scale for Outcomes in Parkinson's disease for Autonomic symptoms (SCOPA-AUT) questions 15–16 (light-headed after standing, fainting), and symptomatic OH prevalence.

To better investigate the effect of LCIG on autonomic symptoms, only AEs possibly related to LCIG, including their severity, were collected for the downstream analysis, while PEG-J placement procedure, surgery, gastrostomy, or device-related AEs were not considered.

### Data analysis

Taking the heterogeneity of both study design and results presentation across reports into consideration, we operated a qualitative evaluation of LCIG efficacy, analyzing significant improvement, stability, or worsening of gastrointestinal, urinary, and cardiovascular symptoms at each timepoint with respect to baseline. Due to the methodological heterogeneity in the assessment and reporting of autonomic outcomes measures in the included studies, we did not perform a meta-analysis. Safety data were evaluated from a quantitative point of view, reporting the total number of AEs not considered to be related to PEG-J placement procedure, surgery, gastrostomy, or device malfunctioning. Results were reported using the mean ± standard deviation (SD), median, or range, as appropriate. Qualitative data were reported using frequency (percentage).

## Results

### Study selection

**Fig. 1:**
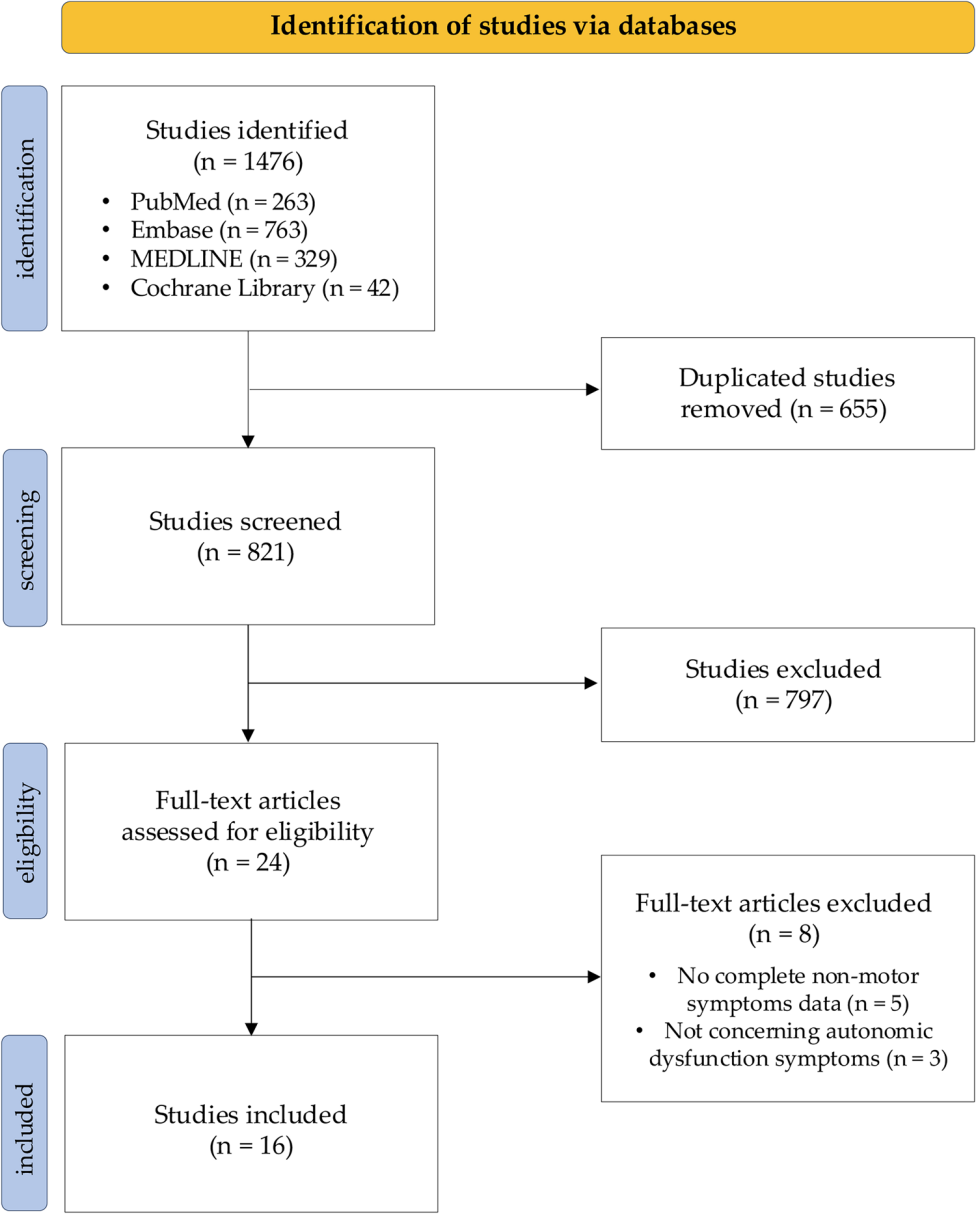
Flowchart of the systematic review process

Of the 1476 studies identified from the databases search, 16 reports/articles met the inclusion criteria and underwent quality assessment and data extraction (Fig. 1), totalling data from 1361 PD individuals (834 men, 527 women). Age and disease duration ranged from 59 to 70 years and from 10 to 15 years, respectively. The average follow-up time was 18.3 months (minimum and maximum follow-up time 2 and 60 months, respectively). To evaluate motor impairment, one study used the MDS-UPDRS-III (mean score 42.5 ± 16), while 15 studies applied the UPDRS-III (scores ranging from 19.1 to 36 points). Twenty-six patients received 24 h of LCIG infusion, whereas in 844 patients the daily infusion duration ranged between 12 and 16 h. Five studies, for a total of 491 patients, did not report total daily hours of LCIG infusion. Twelve studies evaluated autonomic symptoms using the NMSS, and ten studies reported NMSS subdomain scores. Two studies used the MDS-UPDRS part I, while one other study used the SCOPA-AUT (questions 15 and 16). Two studies reported the NMS prevalence, and one study reported NMS severity and frequency. Efficacy and safety data on autonomic symptoms and quality rating with respect to the risk of bias are summarized in Table 1.

**Table 1:** Summary of efficacy and safety data on autonomic symptoms in individuals with advanced PD treated with LCIG

First author of study, year of publication [reference]	Study design	No. of patients	Sex (men %)	Follow-up (months)	Age at LCIG placement	Disease duration at LCIG placement (years)	(MDS)-UPDRS-III at LCIG placement	Rating scale used to assess NMS	Gastrointestinal, urinary and cardiovascular symptoms outcomes	LCIG-related gastrointestinal, urinary and cardiovascular AEs	Quality assessment
Honig et al., 2009 [24]	Prospective, multicenter, open-label, observational study	22	72.7%	6	58.6 ± 9.1	15. ± 5.9	19.1 ± 14	NMSS	A statistically significant beneficial effect was observed for 6 of the 9 NMSS domains (cardiovascular, sleep/fatigue, attention/memory, gastrointestinal, urinary, and miscellaneous) and NMSST. The remaining 3 categories (mood/cognition, perception/hallucinations, sexual function) showed a trend for improvement	Not evaluated	Good
Pursiainen et al., 2012 [32]	Single center, open-label, case series study	9	100%	2	68.5 ± 6.2	12.5 ± 2.2	25 (16—30)	NMSS	Several domains in NMSS (sleep/fatigue, gastrointestinal symptoms, sweating) improved significantly. There was a trend toward lower scores in other domains of NMSS. Sweating or skin temperatures did not change	Not evaluated	Fair
Cáceres-Redondo et al., 2014 [26]	Prospective, observational study	29	43.7%	32 ± 12.4	64.5 ± 9	14.1 ± 3.9	27.2 ± 8.1 (ON) and 48.9 ± 8.9 (OFF)	NMSS	Gastrointestinal tract NMSS domain score was significantly reduced in comparison to baseline at 24 months follow-up evaluation. A non-significant trend for improvement was found for cardiovascular symptoms, including falls, and urinary NMSS domain score	Not reported	Fair
Buongiorno et al., 2015 [22]	Prospective, multicenter, observational study	72	56,9%	48	68.4 ± 7.3/69 (51–87)	13.1 ± 5.1	21.9 (ON), 40.7 (OFF)	UPDRS-I, NMS prevalence	A significant decrease in the prevalence of constipation was reported. No differences were found evaluating other autonomic symptoms	AEs related to treatment were reported in 23 patients. In 3 patients a symptomatic OH was observed, cause of dropout in 1 patient	Poor
Krüger et al., 2017 [40]	Prospective, multicenter, observational study	64	61%	3, 6, 12, > 12	70.4 ± 7.8	13.9 ± 5.4	36 ± 16.2	NMSS	Patients manifested significant improvements in mean change from baseline at every study visit (3, 6 and 12 months) in 5 of 9 NMSS domains: sleep/fatigue, mood/cognition, gastrointestinal tract, urinary and miscellaneous	One-third of patients (32.8%) experienced an AE; 8 patients (12.7%) experienced AEs that were considered to be possibly related to LCIG. Two patients (3.1%) died during the study (cardiac failure and sudden death); both deaths were deemed by the investigator as having no reasonable possibility of being related to LCIG	Good
Standaert et al., 2017 [34]	Open-label phase 3b study	39	59	3, 14	64.3 ± 10.2	11.5 ± 5.3	25 ± 13.2	NMSS	At week 12, urinary and gastrointestinal tract NMSS domain scores were reduced. The latter was maintained at week 60	6 patients with UTI; 5 patients with OH; 1 patient with AF; 1 patient with CCF; 1 patient with internal hernia; 1 patient with peritonitis; 28 patients with any GI-related AEs	Fair
Juhász et al., 2017 [25]	Prospective, multicenter, open-label study from Hungarian registry	34	55.9	12	67 ± 6	12 ± 5	42.5 ± 16	NMSS	Cardiovascular including falls NMSS domain scores were reduced after 12 months in comparison to baseline evaluation. There was no significant change for gastrointestinal tract and urinary NMSS domains score	3 patients with symptomatic OH; 5 patients with weight decrease	Fair
Antonini et al., 2017 [21]	Large multicenter international registry	375	58.7	6, 12, 24	66.4 ± 8.8	12.7 ± 6.3	24.6 ± 12	NMSS	Gastrointestinal tract, cardiovascular including falls and urinary NMSS domains score were reduced at 6, 12 and 18 months of follow-up in comparison to baseline. At 24 months of follow-up these improvements were maintained with the exception of the urinary domain	63 patients with gastrointestinal AEs not procedure or device-related (high prevalence of weight decrease and abdominal pain were reported)	Good
Dafsari et al., 2019 [41]	Prospective, multicenter, observational study	33 LCIG (+ 101 STN-DBS, 39 APO)	51.5%	6	65.4 ± 8.8	14.6 ± 5.3	29.8 ± 12.3	NMSS	Comparison of quality of life, non-motor and motor outcomes among STN-DBS, LCIG, and APO. A statistically significant beneficial effect was noted for gastrointestinal symptoms, sleep, mood/cognition, perceptual problems/hallucinations, attention/memory, and the miscellaneous domains	4 patients reported serious, related to surgery or device (2 patients) or to wound healing disturbance (2 patients); no event related to infusion therapy was reported	Good
Ehlers et al., 2020 [42]	Prospective, open-label study	12	58.3%	6	68 (60–77)	16 (7–27)	21.5 (6–48)	NMSS	Symptoms reported on the NMSS at baseline varied in severity, yet most patients listed problems with sleep/fatigue, mood disorders/cognitive problems, attention/memory, gastrointestinal symptoms, micturition, and miscellaneous problems such as pain or a change in the ability to taste or smell. At follow-up, several subdomains and the overall NMSS score had improved	None of the patients reported any severe adverse events	Fair
Valldeoriola et al., 2021 [43]	Prospective, multicenter, observational study	59	61%	6	67.9 ± 7.5	12.7 ± 6.0	30.1 ± 14.2	NMSS	NMS improved after 6 months of treatment with LCIG. Changes in NMSS scores, both total score and all domain scores, were significantly improved from baseline to the final visit. Sleep/fatigue and gastrointestinal domains were the most improved domains	8 AEs were related to LCIG (ventricular tachycardia, gastrointestinal ulcer, pneumoperitoneum, paralytic ileus, infection, peripheral sensory neuropathy, and substance-induced psychotic disorder)	Good
Standaert et al., 2021 [17]	Subanalysis of DUOGLOBE: multicenter, multinational prospective, real-world, observational	195	61.5%	3, 6, 12	70.2 ± 8.2	11.2 ± 4.8	27.6 ± 13.2	NMSS	Significant reduction in NMSS domain score for cardiovascular including falls, gastrointestinal tract and urinary at each follow-up (3, 6, and 12 month) with exception of urinary NMSS domain at the 12 month follow-up visit	56 patients with weight decrease; 1 patient with UTI; 1 patient with urosepsis; 3 patients with abdominal pain. AEs not meeting criteria for serious AEs were not recorded	Good
Fasano et al., 2021 [11]	Multinational, retrospective, cross-sectional, post-marketing observational study investigating comedication use with LCIG	409	65.3%	12	66.5 ± 7.8	12.8 ± 5.4	30.1 ± 15.3	NMS prevalence	The percentage of patients who experienced NMS of anxiety, pain, depression, and constipation decreased significantly from the initiation of LCIG to the patient visit, whereas the percentage of patients who experienced cognitive impairment, apathy, fatigue, urinary symptoms, and OH increased significantly over that time	AEs likely related to study treatment occurred in 112 patients (27.4%) during LCIG treatment. The most common AEs were stoma infections, dyskinesia, device malfunction, neuropathies, weight decrease	Poor
Stanková et al., 2022 [12]	Prospective interventional study	9 LCIG (+ 8 OMT)	88.9% LCIG—87.5% OMT	6	LCIG 68.44 ± 5.897—OMT 66.125 ± 5.410	10.33 ± 1.9	NR	SCOPA-AUT (questions 15–16), UPDRS-I (1.12)	Symptoms of OH were significantly alleviated in patients after 6 months of LCIG treatment compared to those on OMT	Not evaluated	Good
Chaudhuri et al., 2023 [16]	Multicenter, single- arm, non-interventional, post-marketing, observational study (DUOGLOBE)	See Standaert et al., 2021 [17]	See Standaert et al., 2021 [17]	3, 6, 12, 18, 24, 30, 36	See Standaert et al., 2021 [17]	See Standaert et al., 2021 [17]	See Standaert et al., 2021 [17]	NMSS	NMSS total scores significantly improved from baseline to month 36. In addition, 3 of 9 NMSS subdomains were significantly improved through months 36: sleep/fatigue, gastrointestinal tract and miscellaneous	Safety was assessed by monitoring serious adverse events (SAEs). A total of 107 (54.9%) patients experienced SAEs, with 31 SAEs considered as having a reasonable possibility of being related to LCIG treatment. The most common SAEs were fall (*n* = 8), worsening of PD (*n* = 8), urinary tract infection (*n* = 7)	Good
Fasano et al., 2023 [18]	Multinational, retrospective, cross-sectional post-marketing observational study in patient with APD treated with LCIG in routine clinical care (COSMOS)	See Fasano et al., 2021 [11]	61.6%	12, 24, 36, 48, 60, > 60	67.9 ± 7.4 (12–24 months), 66.2 ± 8.4 (24–36 months), 65.8 ± 7.5 (36–48 months), 64.5 ± 7.6 (48–60 months), 65.0 ± 8.2 (> 60 months)	16.6 ± 3.9 (12–24 months), 30.3 ± 3.3 (24–36 months), 41.8 ± 3.7 (36–48 months), 53.7 ± 3.3 (48–60 months), 78.8 ± 18.6 (> 60 months)	See Fasano et al., 2021 [11]	NMS severity and frequency	Patients had reductions from baseline in the severity and frequency of some of the individual NMS (anxiety, pain, depression, fatigue, constipation). Severity and frequency of patients who experienced cognitive impairment, apathy, fatigue, urinary symptoms, and OH increased over that time	A total of 109 AEs of any type were recorded during LCIG initiation and LCIG maintenance treatment for 387 patients, ranging across groups from 24% in at 12–24 months to 36% at 36–48 months. The most common AEs reported were stoma site infection and unintentional medical device removal	Fair

*AEs* Adverse events, *AF* atrial fibrillation,* APO* apomorphine infusion, *CCF* congestive Cardicac failure, *LCIG* levodopa-carbidopa intestinal gel,* (MDS)-UPDRS* (Movement Disorders Society) Unified Parkinson's Disease Rating Scale, *NMS* non-motor symptoms, *NMSS* Non-Motor Symptoms Scale, *NMSST* Non-Motor Symptoms Scale total score, *PD* Parkinson’s disease, *OH* orthostatic hypotension, *OMT* optimized medical treatment, *SCOPA-AUT* Scales for Outcomes in Parkinson’s disease—Autonomic Dysfunction,* STN-DBS* subthalamic nucleus deep brain stimulation,* UTI* urinary tract infection

### Efficacy

We identified 15 studies reporting the effect of LCIG on gastrointestinal symptoms in a total of 1352 patients. Improvement was reported in four of four studies at T1 (100%), six of seven studies at T2 (85.7%), four of five studies at T3 (80%), three of three studies at T4 (100%), two of two studies at T5 (100%), and one of one study at T6 (100%). Stability was reported in one of seven studies at T2 (14.3%) and one of five studies at T3 (20%). No study reported on worsening of gastrointestinal symptoms. Efficacy data on gastrointestinal symptoms are summarized in Electronic Supplementary Material (ESM) Table 1.

Regarding urinary symptoms, we identified 15 studies involving a total of 1352 patients. Improvement was reported in three of four studies at T1 (75%), six of seven studies at T2 (85.7%), and two of five studies at T3 (40%), while no improvement was reported at T4, T5, and T6. Stability of urinary symptoms under LCIG therapy was reported in one of four studies at T1 (25%), one of seven studies at T2 (14.3%), two of five studies at T3 (40%), three of three studies at T4 (100%), two of two studies at T5 (100%), and one of two studies at T6 (50%). Worsening of urinary symptoms was found in one of five studies at T3 (20%) and one of two studies at T6 (50%) [11, 18]. Efficacy data on urinary symptoms are summarized in ESM Table 2.

Regarding cardiovascular symptoms, we identified 16 studies reporting findings, involving a total of 1361 patients. Improvement of orthostatic intolerance was reported in two of four studies at T1 (50%), five of ten studies at T2 (50%), three of five studies at T3 (60%), and one of three studies at T4 (33.3%), while no improvement was reported at T5 and T6. Stability was reported in two of four studies at T1 (50%), five of ten studies at T2 (50%), one of five studies at T3 (20%), two of three studies at T4 (66.6%), two of two studies at T5 (100%), and one of one study at T6 (100%). One study (20%) reported an increased prevalence of symptomatic OH at T3 [11]. Efficacy data on cardiovascular symptoms are summarized in ESM Table 3.

**Fig. 2:**
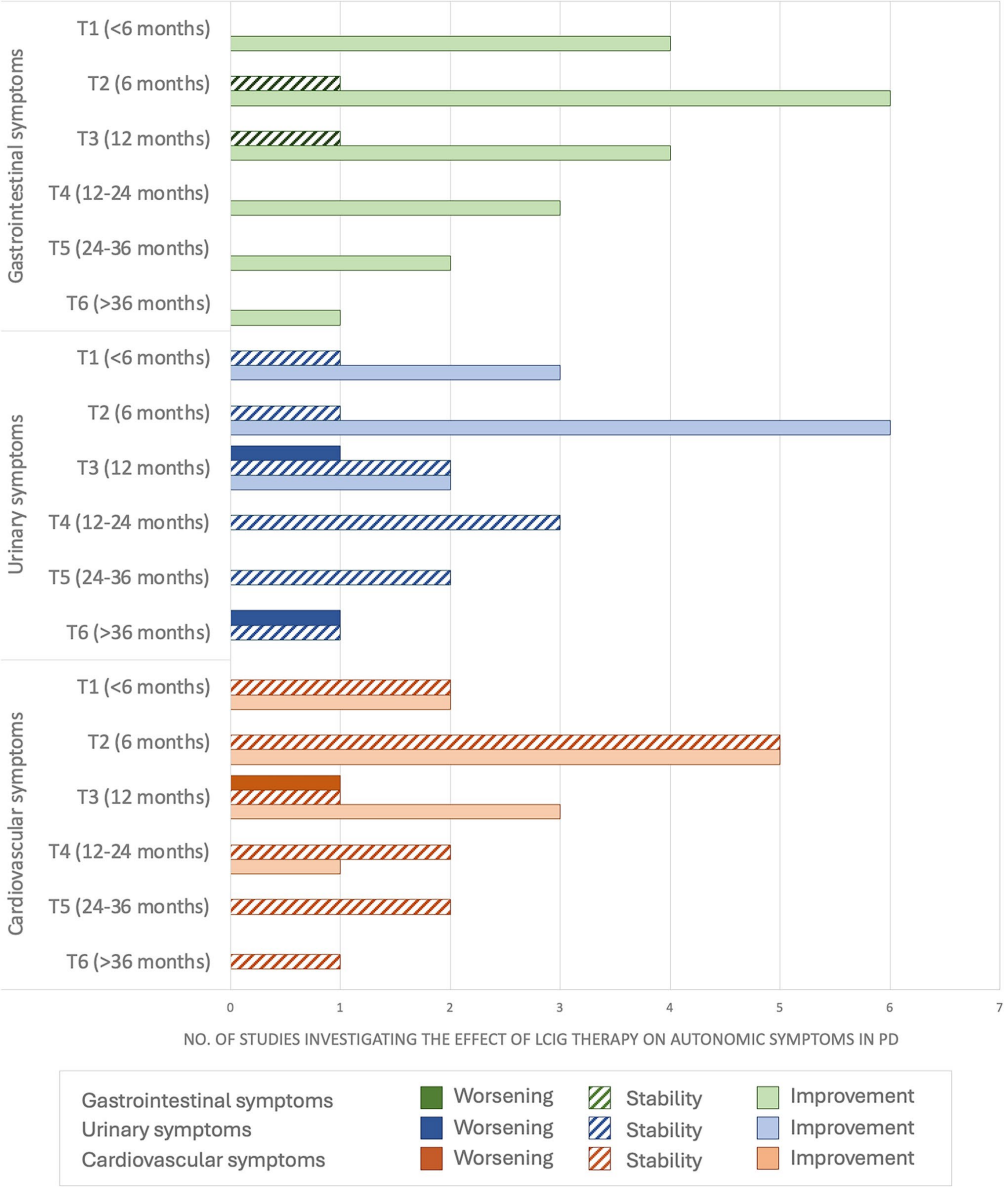
Number of studies reporting improvement, stability, or worsening of gastrointestinal, urinary, and cardiovascular autonomic symptoms at each timepoint. *T Timepoint*

The number of studies reporting improvement, stability or worsening of gastrointestinal, urinary, and cardiovascular autonomic symptoms at each timepoint are summarized in Fig. 2.

### Adverse events and safety

Ten studies analyzed LCIG-related AEs during the entire observation period. Three studies did not collect LCIG-related AEs, and two studies did not clearly expose AEs. Seven studies (43.7%) reported LCIG-related AEs in a total of 1292 patients. There were seven types of gastrointestinal AEs in 109 patients (109/1292, 8.4%) (weight decrease, ulcers, paralytic ileus, internal hernia, peritonitis, abdominal pain, and miscellaneous), one type of urinary AE in seven patients (7/1292, 0.5%) (urinary tract infection), and four types of cardiovascular AEs reported in 14 patients (14/1292, 1.1%) (OH, atrial fibrillation, congestive cardiac failure, ventricular tachycardia). The details regarding the reported AEs are presented in Table 2.

**Table 2:** Summary of gastrointestinal, urinary and cardiovascular adverse events related to LCIG treatment in individuals with advanced PD

First author of study, year of publication [reference]	Gastrointestinal AEs		Urinary AEs		Cardiovascular AEs	
	No. of patients with AE (%)	AEs (no. of patients)	No. of patients with event (%)	AEs (no. of patients)	No. of patients with event (%)	AEs (no. of patients)
Honig et al., 2009 [24]	NA	NA	NA	NA	NA	NA
Pursiainen et al., 2012 [32]	NA	NA	NA	NA	NA	NA
Cáceres-Redondo et al., 2014 [26]	NR	NR	NR	NR	NR	NR
Buongiorno et al., 2015 [22]	0 (0)	–	0 (0)	–	3 (4.1)	Symptomatic OH (3)
Krüger et al., 2017 [40]	0 (0)	–	0 (0)	–	0 (0)	–
Standaert et al., 2017 [34]	30 (76.9)	GI-related AEs (28), peritonitis (1), internal hernia (1)	6 (15.4)	UTI (6)	7 (17.9)	OH (5), AF (1), CCF (1)
Juhász et al., 2017 [25]	5 (14.7)	WD	0 (0)	–	3 (8.8)	Symptomatic OH (3)
Antonini et al., 2017 [21]	63 (16.8)	WD, AP, other GI disorders	0 (0)	–	0 (0)	–
Dafsari et al., 2019 [41]	0 (0)	–	0 (0)	–	0 (0)	–
Ehlers et al., 2020 [42]	0 (0)	–	0 (0)	–	0 (0)	–
Valldeoriola et al., 2021 [43]	2 (3.4)	GI ulcer (1), PI (1)	0 (0)	–	1 (1.7)	VT (1)
Standaert et al., 2021 [17]	see Chaudhuri et al., 2023 [16]		see Chaudhuri et al., 2023 [16]		see Chaudhuri et al., 2023 [16]	
Fasano et al., 2021 [11]	5 (1.2)	WD (5)	0 (0)	–	0 (0)	–
Stanková et al., 2022 [12]	NA	NA	NA	NA	NA	NA
Chaudhuri et al., 2023 [16]	4 (2.1)	AP (4)	1 (0.5)	UTI (1)	0 (0)	–
Fasano et al., 2023 [18]	NR	NR	NR	NR	NR	NR

*AEs* Adverse events, *AF* atrial fibrillation, *AP* abdominal pain, *CCF* congestive cardiac failure, *GI* gastrointestinal, *NA* not available, *NR* not reported, *OH* orthostatic hypotension, *PI* paralytic ileus, *UTI* urinary tract infection, *VT* ventricular tachycardia, *WD* weight decrease

## Discussion

Our systematic assessment indicates an overall improvement or stabilization of gastrointestinal, urinary, and cardiovascular autonomic symptoms in individuals with advanced PD under stable LCIG therapy. Given the well-known detrimental effect of autonomic symptoms on QoL, activities of daily living (ADL), and instrumental activities of daily living (iADL) in PD individuals [20], the pinpointed clinical improvement may ultimately turn into a functional daily benefit.

**Fig. 3:**
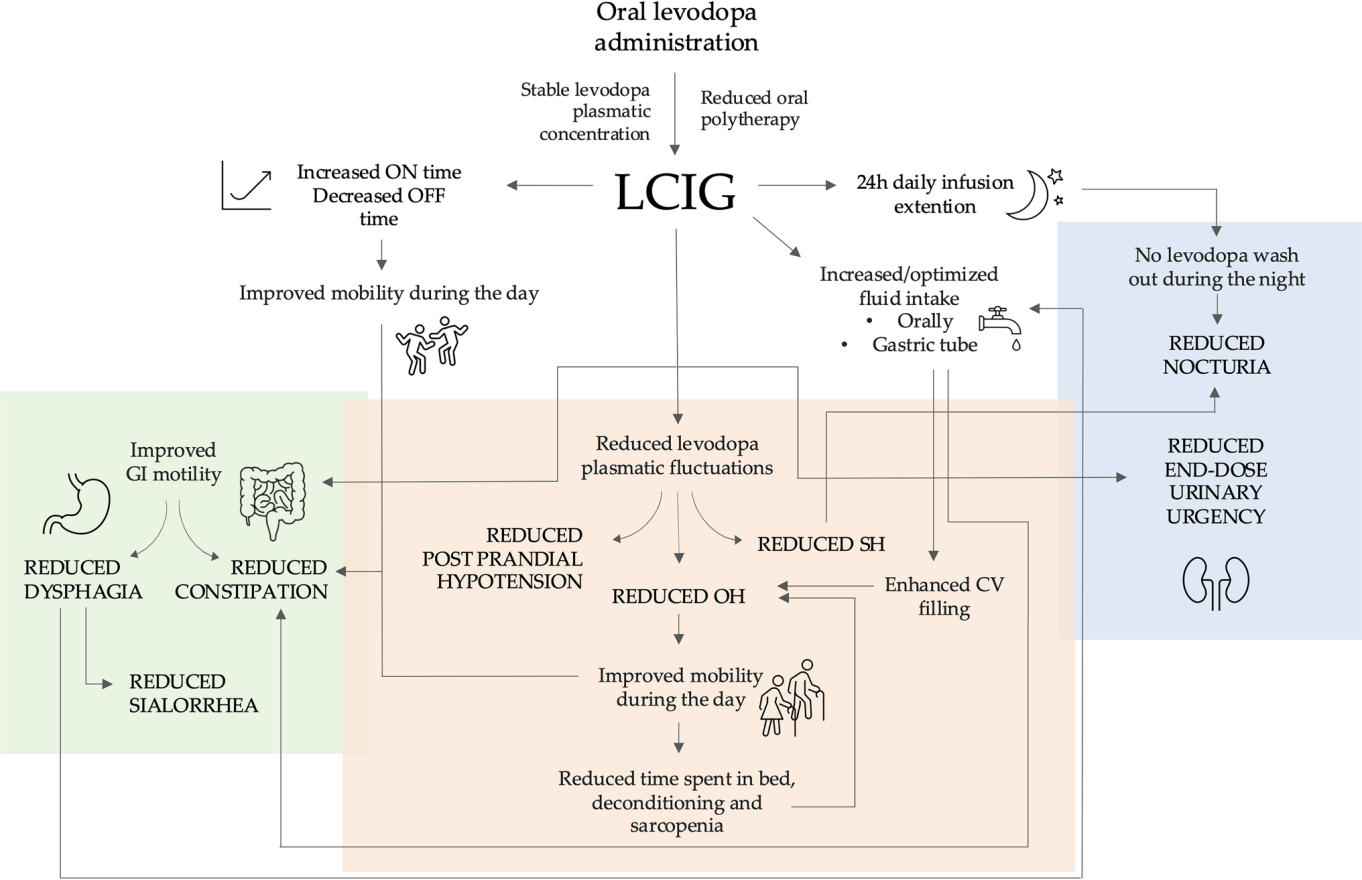
Putative mechanisms underlying the beneficial effects of LCIG on autonomic symptoms in PD. *CV* cardiovascular,* GI* Gastrointestinal, *LCIG* levodopa-carbidopa intestinal gel, *OH* orthostatic hypotension, *SH* supine hypertension,

Studies indicate that the switch from conventional oral treatment to LCIG is accompanied by a significant reduction in the severity, frequency, and duration of motor fluctuations [21]. Non-motor symptoms, including autonomic ones, may equally benefit from a switch to LCIG for multiple reasons [22]. Firstly, switching to LCIG may contribute to ameliorating NMS by reducing the side effects of oral polypharmacy and/or plasma (and brain) peak levodopa concentrations following multiple daily oral administration. Secondly, reducing “OFF” phases may improve the baroreflex sensitivity, as it has been shown that heart-rate variability decreases in PD individuals experiencing freezing of gait during “OFFs” [23]. The reduction of motor fluctuations and dyskinesia might also contribute per se to reducing NMS, as suggested by Honig and colleagues [24], who found an association between UPDRS-IV score improvement and NMSS score reduction in patients following the switch to LCIG therapy. Putative mechanisms on how the benefit offered by LCIG on single parkinsonian symptoms may also positively influence others are summarized in Fig. 3.

In our analysis, only two studies reported worsening of urinary and cardiovascular symptoms [11, 18]. Considering the long observation period in these two studies, however, it is conceivable that the symptomatic worsening was due to PD progression itself, or to any other pharmacological change that may have been implemented over time, rather than to a direct effect of the LCIG regimen.

### Gastrointestinal symptoms

Treatment with LCIG ameliorated gastrointestinal symptoms in all studies, except for two studies which reported stability of symptoms [24, 25]. No study reported worsening of gastrointestinal symptoms. These data suggest the long-term (≥ 24 months) efficacy of LCIG in improving both upper and lower gastrointestinal symptoms [16, 22, 26], likely related to more stable levodopa plasmatic concentrations and less fragmented gastrointestinal motility.

Labeit and colleagues endoscopically evaluated the swallowing process in PD patients after the switch to LCIG and showed a beneficial effect on pharyngeal dysphagia [27], probably due to an overall motor improvement and reduction of motor fluctuations [27]. An improved swallowing ability reduces sialorrhea and facilitates fluid intake; this has in turn the potential to improve both constipation and OH [28]. Improving swallowing also has specific prognostic implications, given the detrimental impact of dysphagia on mortality in PD [29, 30].

Beyond a direct improvement of gastrointestinal motility, improved physical activity during the daytime may equally contribute to amelioration of gastrointestinal transit and constipation. The continuous gel infusion used for LCIG may further soften fecal material, facilitating intestinal transit in a manner similar to osmotic laxatives, and contribute to the long-term benefit on constipation observed in the LCIG-treated PD patients [16, 18].

### Urinary symptoms

Urinary symptoms were shown to benefit from LCIG mainly in the midterm. As already suggested for gastrointestinal symptoms, switching from pulsatile to continuous dopaminergic stimulation may limit the occurrence and severity of levodopa end-of-dose side effects on the autonomic nervous system, such as urinary urgency [28]. Overnight levodopa wash-out under standard oral medications may cause urinary storage symptoms, such as frequent nocturia episodes [28]. Kovács and colleagues reported a significantly different benefit on urinary symptoms in patients treated with LCIG for 24 h than the standard 16 h, supporting the hypothesis that continuous dopaminergic therapy may help to stabilize bladder detrusor overactivity [24, 28, 31].

As mentioned above, urinary symptoms worsened in two studies over the interventional period [11, 18]. We postulate that this might have reflected the progression of PD itself or of other non-neurogenic factors affecting bladder continence and emptying, including non-dopaminergic pharmacological adaptations.

### Cardiovascular symptoms

All but one of the analyzed studies showed improvement or stability of cardiovascular symptoms at all timepoints, especially at the midterm ones.

This could indicate a time-dependent effect of LCIG in improving cardiovascular symptoms or an increased peripheral levodopa tolerance over time [32]. It has been hypothesized that LCIG continuous infusion might improve OH by limiting peak of dose levodopa blood concentrations, in turn reducing the severity of BP fluctuation and postprandial hypotension [12, 25]. The improvement of cardiovascular symptoms could be also related to an overall amelioration in mobility and daily routine, in turn preventing deconditioning, blood venous pooling, and dysphagia-related dehydration [12]. Optimizing fluid intake both orally and through the gastric tube of the PEG-J may also contribute to maintaining an euvolemic state in advanced PD individuals treated with LCIG, thus providing an additional therapeutic tool for cardiovascular autonomic failure [12, 28].

Pursainen and colleagues observed a significant decrease in supine BP of patients in the first 2 months after the switch to LCIG from oral medications [32]. SH is an known risk factor for nocturnal polyuria due to augmented pressure natriuresis [28]. The potential beneficial effect of LCIG on SH could therefore also contribute to reduce sleep fragmentation due to frequent nocturnal toilet visits. All these aspects are in fact a crucial part of the non-pharmacological management of cardiovascular autonomic failure in PD [2, 12].

### Safety data

Regarding safety, an overall quantitative assessment of AEs was hampered by the different methodologies used in the studies. Several aspects must be taken into account in any assessment of AEs, which could limit their evaluation, including a difficult differentiation between LCIG infusion and surgical procedure/PEG-J related AEs, as well as AEs related to treatment and disease progression. An underestimation in reporting AEs has to ultimately be considered.

Commonly observed LCIG-related AEs include dyskinesia, polyneuropathy, confusion, hallucinations, psychosis, and weight loss [33]. PEG-J, surgery, and procedure-related AEs were the most frequently reported AEs in clinical trials, including wound/stoma infection, abdominal/procedural pain, erythema, decubitus ulcer, ileus, and tube dislocation [14].

Our analysis found that only a minority of patients experienced AEs related to LCIG, the majority of which were gastrointestinal, reported by 8% of patients. Although those symptoms were reported as LCIG-related by the authors of the studies, we could not exclude an influence of PEG-J tube presence or surgical placement in their occurrence. AEs related to gastrointestinal autonomic dysfunction per se likely accounted only for part of the reported AEs. Regarding urinary AEs, only seven patients reported a urinary tract infection [17, 34] and 11 developed symptomatic OH (0.8%), ultimately indicating a good safety profile in terms of autonomic symptoms [22, 25, 34]. We could not determine if any LCIG therapy was discontinued due to severe autonomic AE; however the discontinuation rate was overall low across treated patients [15].

### Limitations of the analysis

Our study has a number of limitations. Firstly, we did not consider sexual symptoms as such analyses are complicated by the presence of various contributing factors in older men with PD (e.g., medication side effects, concomitant metabolic, neuropsychiatric, and cardiovascular diseases). Sexual problems are often also poorly explored in female individuals with PD [35, 36]. We also did not focus on thermoregulatory disturbances because most clinical scales used in the reviewed studies included a very limited assessment of thermoregulatory disturbances (i.e., single question on sweating dysfunction in the NMSS) [2].

The heterogeneity in study design, primary and secondary endpoints, sample size, follow-up duration, and clinical methods used to assess NMS may have partially affected our results and ultimately precluded a meta-analysis of the collected data. The lack of standardized quantitative autonomic testing in all but one analyzed study may have also introduced an assessment bias, especially regarding patients with oligo- or asymptomatic autonomic failure [32]. Nevertheless, our results are based on a qualitative data analysis derived from validated and dedicated rating scales widely used to assess PD NMS in clinical and research settings. Given that most of the included studies were primarily designed to assess the impact of LCIG on motor and non-motor symptoms rather than specifically on autonomic ones, a detailed description of non-dopaminergic therapies (including those aimed at controlling autonomic symptoms or potentially exacerbating them) and patient comorbidities was often lacking. Only Pursiainen et al. [32] reported the number of patients with cardiovascular comorbidities and anti-hypertensive therapies, with neither changing throughout the observation period.

Although most studies [21, 22, 24, 26, 41–43] reported variations in levodopa equivalent daily dose (LEDD) before and after LCIG placement, as well as during follow-up, only one study [12] investigated the association between changes in LEDDs and OH symptoms, ultimately reporting no correlation between the two. The interplay between LEDD and changes in severity of parkinsonian NMS over time should be ultimately thoroughly investigated in future studies. The studies included in our review also did not differentiate NMS severity between the “OFF” and “ON” conditions, but rather investigated changes in NMS severity over long time frames (generally focusing on the month prior to the study visit). Rating scales designed explicitly for investigating non-motor fluctuations, such as the Non-Motor-Fluctuations-Assessment [37], possibly combined with the use of wearable devices for remote measurement of cardiovascular parameters and sleep, may help to improve our current understanding of the impact of therapy-dependent autonomic fluctuations on the symptomatic burden of advanced PD [38].

## Conclusions

To our knowledge, this is the first systematic review evaluating the long-term efficacy and safety of LCIG therapy on symptoms of autonomic failure in individuals with advanced PD. The results indicate an overall stability or rather beneficial effect of LCIG on gastrointestinal, urinary and cardiovascular autonomic symptoms,  ultimately broadening the potential advantage of LCIG therapy beyond an improved motor control. They also underscore the importance of considering NMS when choosing the most suitable device-aided therapy for advanced PD cases [39].

## Supplementary Information

Below is the link to the electronic supplementary material.

Supplementary file1 (DOCX 79 KB)
