# Supplementary material for: Effects of levodopa/carbidopa intestinal gel infusion on autonomic symptoms in advanced Parkinson’s disease: a systematic review
Source: Clin Auton Res. 2024 Dec 4;35(2):159–82. doi: 10.1007/s10286-024-01090-9 (PMC12000123; doi:10.1007/s10286-024-01090-9)
Supplement: Supplementary file 1 — Supplementary file1 (DOCX 79 KB) [file 10286_2024_1090_MOESM1_ESM.docx]

***Supplementary Table 1.*** Summary of efficacy data on gastrointestinal symptoms in advanced PD patients treated with LCIG.

| **Author, year** | **Rating scale used for NMS assessment** | **Baseline NMS assessment** | **Baseline NMSS gastrointestinal subdomain** | **Score change**  **at T1 (<6 months)** | **Score change**  **at T2 (6 months)** | **Score change**  **at T3 (12 months)** | **Score change**  **at T4 (12-24 months)** | **Score change at T5 (24-36 months)** | **Score change at T6 (>36 months)** |
| --- | --- | --- | --- | --- | --- | --- | --- | --- | --- |
| Honig et al., 2009 [24] | NMSS, NMSS subdomains | 89.9 ± 56.5 | 10.0 ± 9.3 | NA | 3.8 ± 5.4^a^ | NA | NA | NA | NA |
| Pursiainen et al., 2012 [32] | NMSS, NMSS subdomains | 58 ± 43.81 | 5 (3-11) | 1 (1-4)^a^ | NA | NA | NA | NA | NA |
| Cáceres-Redondo et al., 2014 [26] | NMSS | 17.3 ± 4.7 | NA | NA | NA | NA | NA | NR^a^ | NA |
| Buongiorno et al., 2015 [22] | NMS prevalence | NR | NA | NA | NA | NA | NA | NA | NR^a^ |
| Krüger et al., 2017 [40] | NMSS, NMSS subdomains | 95.5 ± 54.5 | NR | -5.7 ± 6.4^a,d^ | -4.4 ± 6.7^a,d^ | -3.8 ± 6.4^a,d^ | NA | NA | NA |
| Standaert et al., 2017 [34] | NMSS, NMSS subdomains | 48.3 ± 35.6 | 5.3 ± 6.1 | 3.3 ± 5.5^a^ | NA | NA | 3.4 ± 6.8^a^ | NA | NA |
| Juhász et al., 2017 [25] | NMSS, NMSS subdomains | 88.9 ± 40.3 | 8.4 ± 7.1 | NA | NA | 6.5 ± 7.8^b^ | NA | NA | NA |
| Antonini et al., 2017 [21] | NMSS, NMSS subdomains | 69.2 ± 42.1 | 7.5 ± 6.8 (227 pz) | NA | -2.5 ± 6.9^a,d^ | -2.5 ± 6.9^a,d^ | -2.7 ± 6.9^a,d^ | NA | NA |
| Dafsari et al., 2019 [41] | NMSS, NMSS subdomains | 86.9 ± 45.5 | 10.5 ± 9.2 | NA | 7.5 ± 8.2^a^ | NA | NA | NA | NA |
| Ehlers et al., 2020 [42] | NMSS, NMSS subdomains | 93 (21-164) | 3 (0-20) | NA | 3.5 (0-12)^b^ | NA | NA | NA | NA |
| Valldeoriola et al., 2021 [43] | NMSS, NMSS subdomains | 83.2 ± 32.6 | 7.4 | NR | 3.5^a^ | NA | NA | NA | NA |
| Standaert et al., 2021 [17] | NMSS, NMSS subdomains | 87.9 ± 51.3 | 9.5 ± 8.2 | -3.7 ± 6.2^a,d^ | -4 ± 7.5^a,d^ | -3.2 ± 7.7^a,d^ | NA | NA | NA |
| Fasano et al., 2021 [11] | NMS prevalence | NR | NR | NA | NA | NR^a^ | NA | NA | NA |
| Chaudhuri et al., 2023 [16] | NMSS | 88.2 ± 51.1 | 9.5 ± 8.2 | see Standaert et al., 2021 | see Standaert et al., 2021 | see Standaert et al., 2021 | NR^a^ | NR^a^ | NA |
| Fasano et al., 2023 [18] | NMS severity and frequency | NR | NR | NA | NA | NR | NR | NR | NR |

LCIG: Levodopa-carbidopa intestinal gel; PD: Parkinson’s disease; AEs: Adverse events; NMS: Non-motor symptoms; NMSS: Non-motor symptoms scale.

NA: Not available

NR: Not reported

^a^ Significant improvement

^b^ No significant improvement or worsening

^c^ Significant worsening

^d^ Change from baseline

***Supplementary Table 2***. Summary of efficacy data on urinary symptoms in advanced PD patients treated with LCIG.

| **Author, year** | **Rating scale used for NMS assessment** | **Baseline NMS assessment** | **Baseline NMSS urinary subdomain** | **Score change**  **at T1 (<6 months)** | **Score change**  **at T2 (6 months)** | **Score change**  **at T3 (12 months)** | **Score change**  **at T4 (12-24 months)** | **Score change at T5 (24-36 months)** | **Score change at T6 (>36 months)** |
| --- | --- | --- | --- | --- | --- | --- | --- | --- | --- |
| Honig et al., 2009 [24] | NMSS, NMSS subdomains | 89.9 ± 56.5 | 2.9 ± 3.6 | NA | 4,8 ± 6.1^a^ | NA | NA | NA | NA |
| Pursiainen et al., 2012 [32] | NMSS, NMSS subdomains | 58 ± 43.81 | 0 (0-3) | 6 (0-9)^b^ | NA | NA | NA | NA | NA |
| Cáceres-Redondo et al., 2014 [26] | NMSS | 17.3 ± 4.7 | NR | NA | NA | NA | NA | NR^b^ | NA |
| Buongiorno et al., 2015 [22] | NMS prevalence | prevalence = 58% | NR | NA | NA | NA | NA | NA | prevalence = 36%^b^ |
| Krüger et al., 2017 [40] | NMSS, NMSS subdomains | 95.5 ± 54.5 | NR | -7.4 ± 10.7^a,d^ | -5.9 ± 10.5^a,d^ | -5.9 ± 9.9^a,d^ | NA | NA | NA |
| Standaert et al., 2017 [34] | NMSS, NMSS subdomains | 48.3 ± 35.6 | 1.4 ± 2.1 | 6.1 ± 7.3^a^ | NA | NA | 8.4 ± 9.6^b^ | NA | NA |
| Juhász et al., 2017 [25] | NMSS, NMSS subdomains | 88.9 ± 40.3 | 5.4 ± 3.2 | NA | NA | 10.7± 7.8^b^ | NA | NA | NA |
| Antonini et al., 2017 [21] | NMSS, NMSS subdomains | 69.2 ± 42.1 | 2.4 ± 3.3 | NA | -2.1 ± 9.1^a,d^ | -2.1 ± 9.1^a,d^ | -0.7 ± 11.2^b,d^ | NA | NA |
| Dafsari et al., 2019 [41] | NMSS, NMSS subdomains | 86.9 ± 45.5 | 3.6 ± 5.2 | NA | 9.4 ± 9.3^b^ | NA | NA | NA | NA |
| Ehlers et al., 2020 [42] | NMSS, NMSS subdomains | 93 (21-164) | 0 (0-12) | NA | 4 (0-26)^a^ | NA | NA | NA | NA |
| Valldeoriola et al., 2021 [43] | NMSS, NMSS subdomains | 83.2 ± 32.6 | 2.5 | NA | 7.4^a^ | NA | NA | NA | NA |
| Standaert et al., 2021 [17] | NMSS, NMSS subdomains | 87.9 ± 51.3 | 14.3 ± 11.0 | -2,7 ± 9.2^a,d^ | -2.5 ± 9.5^a,d^ | -1.6 ± 10.5^b,d^ | NA | NA | NA |
| Fasano et al., 2021 [11] | NMS prevalence | NR | NR | NA | NA | NR^c^ | NA | NA | NA |
| Chaudhuri et al., 2023 [16] | NMSS | 88.2 ± 51.1 | 14.3 ± 11.0 | see Standaert et al., 2021 | see Standaert et al., 2021 | see Standaert et al., 2021 | NR^b^ | NR^b^ | NA |
| Fasano et al., 2023 [18] | NMS severity and frequency | NR | NR | NA | NA | NR | NR | NR | NR^c^ |

LCIG: Levodopa-carbidopa intestinal gel; PD: Parkinson’s disease; AEs: Adverse events; NMS: Non-motor symptoms; NMSS: Non-motor symptoms scale.

NA: Not available

NR: Not reported

^a^ Significant improvement

^b^ No significant improvement or worsening

^c^ Significant worsening

^d^ Change from baseline

***Supplementary Table 3.*** Summary of efficacy data on cardiovascular symptoms in advanced PD patients treated with LCIG.

| **Author, year** | **Rating scale used for NMS assessment** | **Baseline NMS assessment** | **Baseline NMSS cardiovascular subdomain** | **Score change at T1 (<6 months)** | **Score change at T2 (6 months)** | **Score change at T3 (12 months)** | **Score change at T4 (12-24 months)** | **Score change at T5 (24-36 months)** | **Score change at T6 (>36 months)** |
| --- | --- | --- | --- | --- | --- | --- | --- | --- | --- |
| Honig et al., 2009 [24] | NMSS, NMSS subdomains | 89.9 ± 56.5 | 2.9 ± 3.6 | NA | 0.5 ± 1.3^a^ | NA | NA | NA | NA |
| Pursiainen et al., 2012 [32] | NMSS, NMSS subdomains | 58 ± 43.81 | 0 (0-3) | 0 (0-8)^b^ | NA | NA | NA | NA | NA |
| Cáceres-Redondo et al., 2014 [26] | NMSS | 17.3 ± 4.7 | NR | NA | NA | NA | NA | NR^b^ | NA |
| Buongiorno et al., 2015 [22] | NMS prevalence | NR | NA | NA | NA | NA | NA | NA | NR^b^ |
| Krüger et al., 2017 [40] | NMSS, NMSS subdomains | 95.5 ± 54.5 | NR | -1.7 ± 4.3^a,d^ | -0.8 ± 3.0^b,d^ | -0.8 ± 3.3^b,d^ | NA | NA | NA |
| Standaert et al., 2017 [34] | NMSS, NMSS subdomains | 48.3 ± 35.6 | 1.4 ± 2.1 | 1.2 ± 1.7^b^ | NA | NA | 1.9 ± 2.5^b^ | NA | NA |
| Juhász et al., 2017 [25] | NMSS, NMSS subdomains | 88.9 ± 40.3 | 5.4 ± 3.2 | NA | NA | 3.8 ± 5.3^a^ | NA | NA | NA |
| Antonini et al., 2017 [21] | NMSS, NMSS subdomains | 69.2 ± 42.1 | 2.4 ± 3.3 | NA | -0,6 ± 4^a,d^ | -1.1 ± 3.8^a,d^ | -0,8 ± 4^a,d^ | NA | NA |
| Dafsari et al., 2019 [41] | NMSS, NMSS subdomains | 86.9 ± 45.5 | 3.6 ± 5.2 | NA | 2.8 ± 4.4^b^ | NA | NA | NA | NA |
| Ehlers et al., 2020 [42] | NMSS, NMSS subdomains | 93 (21-164) | 0 (0-12) | NA | 0 (0-4)^b^ | NA | NA | NA | NA |
| Valldeoriola et al., 2021 [43] | NMSS, NMSS subdomains | 83.2 ± 32.6 | 2.5 | NA | 0.8^a^ | NA | NA | NA | NA |
| Standaert et al., 2021 [17] | NMSS, NMSS subdomains | 87.9 ± 51.3 | 3.1 ± 4.4 | -1.1 ± 4.2^a,d^ | -1 ± 4.7^a,d^ | -0.8 ± 4.1^a,d^ | NA | NA | NA |
| Fasano et al., 2021 [11] | NMS prevalence | NR | NR | NA | NA | NR^c^ | NA | NA | NA |
| Stanková et al., 2022 [12] | SCOPA-AUT questions 15 | 0.71 ± 0.76 | NA | NA | 0.43 ± 0.53^a^ | NA | NA | NA | NA |
|  | SCOPA-AUT questions 16 | 0.37 ± 0.52 | NA | NA | 0.00 ± 0.00^b^ | NA | NA | NA | NA |
|  | MDS-UPDRS I 1.12 | 1.5 ± 1.07 | NA | NA | 0.87 ± 1.13^b^ | NA | NA | NA | NA |
| Chaudhuri et al., 2023 [16] | NMSS | 88.2 ± 51.1 | 3.1 ± 4.4 | see Standaert et al., 2021 | see Standaert et al., 2021 | see Standaert et al., 2021 | NR^b^ | NR^b^ | NA |
| Fasano et al., 2023 [18] | NMS severity and frequency | NR | NR | NA | NA | NR | NR | NR | NR |

LCIG: Levodopa-carbidopa intestinal gel; PD: Parkinson’s disease; AEs: Adverse events; NMS: Non-motor symptoms; NMSS: Non-motor symptoms scale; SCOPA-AUT: SCales for Outcomes in PArkinson’s disease - Autonomic Dysfunction; MDS-UPDRS: Movement Disorders Society Unified Parkinson Disease Rating Scale.

NA: Not available

NR: Not reported

^a^ Significant improvement

^b^ No significant improvement or worsening

^c^ Significant worsening

^d^ Change from baseline
